# Metaplastic Breast Cancer Characteristics and Patterns of Recurrence: A Single Institution Experience

**DOI:** 10.7759/cureus.101090

**Published:** 2026-01-08

**Authors:** Baraah M Mohamed, Haydee Del Calvo, Ambika Singh, Fatemeh Shojaeian, Nancy Buderer, Olutayo A Sogunro

**Affiliations:** 1 General Surgery, York Wellspan, York, USA; 2 Department of Surgery and Division of Breast Surgical Oncology, Johns Hopkins Hospitals, Baltimore, USA; 3 Medicine, Nova Southeastern University Dr. Kiran C. Patel College of Osteopathic Medicine, Fort Lauderdale, USA; 4 Department of Surgery, Johns Hopkins University School of Medicine, Baltimore, USA; 5 Statistics, Nancy Buderer Consulting, LLC, Ohio, USA; 6 Department of Surgery and Division of Breast Surgical Oncology, Johns Hopkins Hospitals, Maryland, USA

**Keywords:** aggressive breast cancer, clinicopathologic characteristics, keynote-522, metaplastic breast cancer, neoadjuvant chemotherapy, pathologic complete response, recurrence patterns

## Abstract

Background

Metaplastic breast cancer (MtBC) is a rare type of aggressive breast cancer. MtBC has multiple pathological subtypes, including squamous, sarcomatoid, spindle, adenosquamous, and mixed metaplastic. Given the rare nature of the disease, there is limited information available on the behaviour of this cancer and no well-defined recommendations for treatment. We aim to investigate the clinicopathological characteristics of MtBC, including overall survival and patterns of recurrence.

Methods

We conducted a single-institution retrospective review of 91 patients with MtBC who presented from 2009 to 2024. We collected patient-specific, clinicopathological, and survival data. Recurrence-free interval, medical and surgical treatment, follow-up, and time to death were also collected. Data is described with frequency counts and percentages. Product-limit survival estimates were calculated from the time from diagnosis to last follow-up or death.

Results

Our population consisted of 91 patients; the mean age at diagnosis was 60 years. All patients were female; the majority were Caucasian (59, 67.1%), followed by African American (24, 27.3%). Sixty-six patients (72.5%) had a BMI >25, and 69 (78.4%) were postmenopausal. MtBC was estrogen, progesterone, and human epidermal growth factor (Her2) negative in 82 (90.1%), 88 (96.7%), and 80 (88.9%) of the patients, respectively. Forty-five patients (49.5%) underwent a lumpectomy, 41 (45.1%) a mastectomy, and one (1.1%) no surgery. Sixty-four patients (73.6%) had undergone sentinel lymph node biopsy (SLNB), 16 (18.4%) had axillary lymph node dissection (ALND), three (3.4%) had targeted axillary surgery, and four (4.6%) had no axillary surgery. Sixty-six patients (76.7%) underwent chemotherapy, of which 27 (40.9%) were in the neoadjuvant chemotherapy (NACT) setting. Of the patients who underwent NACT, seven had a complete pathological response. Two of the pathologic complete response (pCR) group received Keynote-522. In our population, 29 (36.3%) patients had a recurrence, of which 12 (41.4%) had distant recurrence, with a median time to recurrence of 12 months. The 12-month, 36-month, and 60-month overall survival rates were 93.9% (Standard Error (SE): 2.7%), 81.3% (SE: 4.8%), and 79.2% (SE: 5.1%), respectively. There was no significant difference in the survival rates (p= 0.88) or recurrence rates (p=0.19) for NACT versus adjuvant cases. Subgroup analysis comparing patients with and without recurrence who received NACT revealed no statistically significant differences in clinicopathologic features such as grade, ER, PR, HER2 status, or metaplastic subtype. Moreover, 20 patients in this study received both chemotherapy and immunotherapy, and six (33.3%) experienced recurrences, with no significant differences in recurrence rates, recurrence pattern (p=0.40), or timing (p=0.32) between NACT and adjuvant groups.

Conclusion

We demonstrated that MtBC exhibits aggressive oncologic behaviour, often with triple-negative receptor status and high Ki-67. The most common pattern of recurrence is distant, mainly involving the lungs; hence, a chest tomography was essential during the one-year follow-up to detect these lesions. Seven patients (25.9%) who received NACT achieved a pCR, indicating that NACT can be highly beneficial for a subset of patients. Further studies investigating the effects of NACT, especially keynote-522, in a larger population are necessary to create standardized treatment.

## Introduction

Metaplastic breast cancer (MtBC) is a rare and aggressive subtype of breast carcinoma, accounting for 0.2-1% of breast cancer diagnoses in the United States [1]. First described in 1973, MtBC was officially recognized as a distinct breast cancer subtype by the World Health Organization (WHO) in 2000, highlighting the need for further research into its pathology [2]. Due to its recent classification, many aspects of MtBC remain poorly understood. MtBC is characterized by rapid tumor growth and the histological presence of at least two cellular types, most commonly epithelial and mesenchymal components [3]. MtBC frequently exhibits a triple-negative breast cancer (TNBC) phenotype, lacking expression of the estrogen receptor (ER), human epidermal growth factor 2 receptor (HER2), and progesterone receptor (PR) [3]. The WHO has further classified MtBC into subtypes, including fibromatosis-like, low-grade adenosquamous carcinoma, metaplastic carcinoma with mesenchymal differentiation, mixed metaplastic carcinoma, spindle cell carcinoma, and squamous cell carcinoma [3]. This classification helps identify low-grade and high-grade tumors, enabling optimized treatment strategies. Notably, except for low-grade adenosquamous and fibromatosis-like variants, most MtBC subtypes are highly aggressive and chemotherapy-resistant [4]. 

Unlike other breast cancers, MtBC exhibits less axillary lymph node involvement due to its tendency to spread hematogenously as opposed to spreading lymphatically [1]. Consequently, MtBC more frequently metastasizes to distant organs, contributing to its poor prognosis [5]. In contrast, larger invasive breast cancers show axillary lymph node involvement in over 50% of cases, whereas MtBC's incidence ranges from 6% to 26% [6]. Due to its rapid growth and tendency to spread hematogenously, MtBC has been described as having a poor overall prognosis. This prognosis can be attributed to the early progression of MtBC and its ability to spread to distant organs, such as the lungs and brain [7]. However, this may stem from the lack of standardized diagnostic criteria due to limited available data.

Given these challenges, physicians face significant obstacles in determining the optimal treatment plan for MtBC. Currently, MtBC patients are treated with a combination of chemotherapy, radiation, and surgery, with varying degrees of success [8]. However, neoadjuvant chemotherapy (NACT) has shown promising results in the treatment of MtBC [9]. In one of the case reports, the keynote-522 clinical algorithm using pembrolizumab, paclitaxel, carboplatin, adriamycin, and cyclophosphamide in a neoadjuvant fashion was given to a triple-negative MtBC patient and resulted in a complete pathological response [10]. While these findings are encouraging, a definitive association between the overall survival in MtBC patients and NACT has yet to be established [9]. In this study, we aim to further investigate the clinicopathological characteristics of MtBC, including overall survival and patterns of recurrence, within a single institution. This article was previously presented as a poster at the 26th American Society of Breast Surgeons Annual Meeting on April 30th, 2025.

## Materials and methods

This is a single-institution retrospective review study of patients diagnosed with MtBC from 2009 to 2024. The chart review was conducted in accordance with institutional research policies, and study approval was obtained from the Johns Hopkins School of Medicine Institutional Review Board (IRB)-3 (Howard Lederman) (approval number IRB00470306). All patients aged 18 years and older who presented to our institution and had a biopsy-confirmed diagnosis of MtBC during the defined study period from 2009 to 2024 were included in the analysis. Patients outside the study period were excluded. 

We collected patient-specific, clinicopathological, and survival data. Patient-specific data comprised of age, gender, race, BMI, menopausal status, and genetic mutations. Clinicopathological data consisted of metaplastic type, grade, Ki-67, receptor status, pathological size, staging, laterality, and treatment modalities. Survival data included recurrence-free interval, time to death, type of recurrence, and last date of follow-up. All the durations were measured from the time of biopsy-proven diagnosis. 

Data were described using frequency counts, percentages, mean (standard deviation), or median (interquartile range). Product-limit survival estimates were calculated from the time of diagnosis until last follow-up or death. Group comparisons were made using the Chi-square test, Fisher's Exact test (for small cell sizes), or the Mann-Whitney-Wilcoxon test. All p-values were two-tailed. Data was analyzed using Statistical Analysis System (SAS) software, version 9.4 (SAS Institute Inc., Cary, NC).

## Results

A total of 91 patients diagnosed with MtBC from 2009 to 2024 were included in the study. All the patients were females, with a mean age at diagnosis of 60 years. Among the patients with known ethnicity (n=88), the majority were White (n=59, 67.05%), followed by African Americans (n=24, 27.27%), with few Asian (n=3, 3.41%), Hispanic or Latino (n=1, 1.14%), and other ethnicities (n=1, 1.14%). Of the patients with recorded menopausal status, most were postmenopausal (n=69, 78.41%). At the time of the diagnosis, sixty-six patients (72.52%) had a body mass index (BMI) ≥25. 

Among patients with genetic testing results available (n=58), 49 (84.45%) had no pathogenic mutations. The remaining cases included BRCA1 (n=3, 5.17%), ATM (n=2, 3.45%), BRCA2 (n=1, 1.72%), and P53 (n=1, 1.72%) mutations. The proliferation index, Ki-67, was elevated in our population with a mean value of 57% (SD 26). A summary of the demographics of all the patients is outlined in Table 1. 

**Table 1:** Patients demographics *Patients with missing documentation regarding that specific category are excluded from the percentage calculation for that category.

Patient Characteristics	n (%)
Age of diagnosis, mean (SD)	60 (15)
Female gender	91 (100%)
Ethnicity	
Black or African-American	24 (27.27%)
White or Caucasian	59 (67.05%)
Hispanic or Latino	1 (1.14%)
Asian	3 (3.41%)
Other	1 (1.14%)
Missing	3 *
BMI at diagnosis	
<25	25 (27.47%)
25 – 29	34 (37.36%)
>=30	32 (35.16%)
Menopause	
Pre	19 (21.60%)
Post	69 (78.41%)
Missing	3 *
Known genetic mutation	
None	49 (84.45%)
Others	2 (3.45%)
BRCA1	3 (5.17%)
BRCA2	1 (1.72%)
P53	1 (1.72%)
ATM	2 (3.45%)
Missing	33 *
Ki 67 (%), mean (SD); n=32 missing	57 (26)

Multiple MtBC subtypes were identified, including spindle cell carcinoma (n=18, 19.78%), squamous cell carcinoma (n=17, 18.68%), adenosquamous carcinoma (n=2, 2.20%), and mixed metaplastic (n=9, 9.89%). The remaining (n=45, 49.45%) fell into other smaller categories. Of the tumors with a known grade (n=86), most were grade 3 (n=70, 81.40%). Most tumors were ER-negative (n=82, 90.11%) and PR-negative (n=88, 96.70%). Of the 90 patients with known HER2 status, 88.89% (n=80) were HER2-negative. Using imaging at the time of diagnosis, the average tumor size was about 30 mm (standard deviation = 16). The most common pathological staging among patients with a known stage (n=84) was stage II (n=44, 52.38%), followed by stage I (n=22, 26.19%), stage III (n=9, 10.71%), and stage IV (n=4, 4.76%). The characteristics of all the tumors are summarized in Table 2. 

**Table 2:** Tumor characteristics *Patients with missing documentation regarding that specific category are excluded from the percentage calculation for that category. AJCC: American Joint Committee on Cancer.

Tumor Characteristics	n (%)
Metaplastic subtype * * * *	
Squamous cell carcinoma	17 (18.68%)
Spindle cell carcinoma	18 (19.78%)
Adenosquamous	2 (2.20%)
Mixed metaplastic	9 (9.89%)
Others	45 (49.45%)
Grade* * * *	
1	5 (5.75%)
2	11 (12.64%)
3	70 (81.40%)
Missing	5 *
Estrogen receptor * * * *	
Positive	9 (9.89%)
Negative	82 (90.11%)
Progesterone receptor* * * *	
Positive	3 (3.30%)
Negative	88 (96.70%)
HER2 * * * *	
Positive	10 (11.11%)
Negative	80 (88.89%)
Missing * *	1 *
Lesion on imaging * * * *	
Unifocal	81 (89.01%)
Multifocal	9 (9.89%)
Multicenteric	1 (1.10%)
Positive nodes before any chemotherapy* * * *	
None	64 (79.01%)
1	8 (9.88%)
2	2 (2.47%)
3	1 (1.23%)
4	6 (7.41%)
Missing	10*
Number margins positive* * * *	
None	79 (96.34%)
1	2 (2.44%)
2	1 (1.21%)
Missing or n.a.	9 *
AJCC T status* * * *	
T0	7 (8.14%)
T1	26 (30.23%)
T2	33 (38.37%)
T3	18 (20.93%)
T4	2 (2.32%)
Missing	5 *
AJCC N status* * * *	
N0	65 (81.25%)
N1	11 (13.75%)
N2	4 (5.00%)
Missing	11 *
AJCC M status* *	
M0	84 (95.45%)
M1	4 (4.55%)
Missing	3 *
Pathological stage* *	
0	5 (5.95%)
1	22 (26.19%)
2	44 (52.38%)
3	9 (10.71%)
4	4 (4.76%)
Missing (codes 5 or 6)	7 *
Cancer laterality* *	
Left	56 (61.54%)
Right	25 (27.47%)
Tumor size (mm) imaging, mean (SD), n=1 missing * *	30 (16)
Pathologic size (mm), mean (SD), n=7 missing* *	32 (27)

Treatment modalities varied between patients, including surgery, radiation, and chemotherapy. Of the patients who underwent surgery, 45 patients (49.45%) underwent a lumpectomy, and the remainder had variable mastectomy variants. SLNB was the most common nodal surgery among patients (n=64, 73.56%), excluding patients with missing nodal documentation. Radiation therapy to both the chest and axilla was administered to 51 patients (60.71%), excluding patients with missing documentation for radiation therapy. Of the patients who received chemotherapy (n=66), 27 (40.91%) received it in the neoadjuvant setting and 39 (59.09%) in the adjuvant setting. Immunotherapy was administered to 21 patients (24.42%), and endocrine therapy to 14 patients (16.47%), as exhibited in Table 3. Of the patients who received various regimens of neoadjuvant chemotherapy, seven patients had a complete pathological response.

**Table 3:** Treatment modalities amongst the patient population *Patients with missing documentation regarding that specific category are excluded from the percentage calculation for that category. SLNB: Sentinel lymph node biopsy, AXLD: Axillary lymph node dissection.

Treatment	n (%)
Initial cancer surgery* *	
No surgery	1 (1.1%)
Simple mastectomy	22 (24.18%)
Nipple sparing mastectomy	7 (7.69%)
Skin sparing mastectomy	4 (4.40%)
Modified radical mastectomy	8 (8.79%)
Lumpectomy	45 (49.45%)
Other	4 (4.40%)
Nodal surgery* *	
SLNB	64 (73.56%)
AXLD	16 (18.39%)
Targeted dissection	3 (3.45%)
No axillary surgery	4 (4.60%)
Missing	4 *
Radiation * * * *	
None	30 (35.71%)
Chest adjuvant	3 (3.57%)
Both chest and axilla adjuvant	51 (60.71%)
Missing	7 *
Chemotherapy	
No	20 (23.26%)
Yes	66 (76.74%)
Missing	5*
Timing of Chemotherapy * * * *	
Neoadjuvant	27 (40.91%)
Adjuvant	39 (59.09%)
n.a.	25 *
Immunotherapy* * * *	
No	65 (75.58%)
Yes	21 (24.42%)
Missing	5*
Endocrine therapy* *	
No	70 (82.35%)
Yes	14 (16.47%)
Neoadjuvant	1 (1.18%)
Missing	6*

Eighty patients had clear documentation of recurrence status; recurrences were observed in 29 patients (36.25%), of whom 12 (41.37%) showed a distant pattern. The median time to recurrence was 12 months (interquartile range 8-26 months). Recurrence was high among patients with the squamous cell carcinoma subtype (n=6, 42.86%). Adenosquamous carcinoma had no recorded recurrences in our study. 

Subgroup analysis comparing the presence and absence of recurrence in NACT patients revealed no statistically significant differences in clinicopathologic features such as grade, ER, PR, HER2 status, or metaplastic subtype, as shown in Table 4.

**Table 4:** Characteristics of neoadjuvant cases with and without recurrence a-Chi-square, b-Fisher's Exact *Patients with missing documentation regarding that specific category are excluded from the percentage calculation for that category.

Characteristics	Neoadjuvant With Recurrence (9)	Neoadjuvant Without Recurrence (16)	p-value
Metaplastic subtype, n (%)			
Squamous cell carcinoma	2 (22.22%)	3 (18.75%)	0.95b
Spindle cell carcinoma	2 (22.22%)	2 (12.50%)	
Adenosquamous	0 (0.00%)	1 (6.25%)	
Mixed metaplastic	1 (11.11%)	1 (6.25%)	
Others	4 (44.44%)	9 (56.25%)	
Missing	2*		
Grade, n (%)			
1	0 (0.00%)	0 (0.00%)	0.54b
2	0 (0.00%)	2 (12.50%)	
3	8 (100.00%)	14 (87.5%)	
Missing	3 *		
Estrogen receptor, n (%) * *			
Positive	0 (0.00%)	1 (6.25%)	1.0b
Negative	9 (100.00%)	15 (93.75%)	
Missing	2*		
Progesterone receptor, n (%)* * * *			
Positive	0 (0.00%)	0 (0.00%)	n.a.
Negative	9 (100.00%)	16 (100.00%)	
Missing	2*		
HER2, n (%)* *			
Positive	1 (11.11%)	4 (25.00%)	0.62b
Negative	8 (88.89%)	12 (75.00%)	
Missing	2 *		

Another subgroup analysis of patients who received chemotherapy (n = 66), comparing NACT patients to adjuvant chemotherapy patients, showed that there is no statistically significant difference in recurrence rates between those treated with neoadjuvant (n=9, 36.0%) versus adjuvant chemotherapy (n=13, 37.1%). Both patterns of recurrence (p = 0.31) and time to recurrence (p = 0.19) were also compared, with no significant differences between the two groups, as shown in Table 5.

**Table 5:** Clinical outcomes of chemotherapy cases (Neoadjuvant vs. Adjuvant) *Patients with missing documentation regarding that specific category are excluded from the percentage calculation for that category.

Outcome	Total (66)	Neoadjuvant (27)	Adjuvant (39)	p-value	Chi-square value, χ²
Recurrence, n (%)					
Yes	22 (36.7%)	9 (36.0%)	13 (37.1%)	0.93a	0.008
No	38 (63.3%)	16 (64.0%)	22 (62.9%)		
Missing	6*				
Recurrence pattern, n (%)					
Local	6 (27.3%)	1 (11.1%)	5 (38.5%)	0.31b	-
Regional	0 (0%)	0 (0%)	0 (0%)		
Distant	10 (45.5%)	6 (66.7%)	4 (30.8%)		
Multiple	6 (27.3%)	2 (22.2%)	4 (30.8%)		
N/A	44*				
Median (IQR) time to recurrence, months (among n=22 recurrences)	16.5 (9,33)	12 (9,17)	26 (12,36)	0.19c	-
Range, months	1.5-108	6-33	1.5-108		
a-Chi-square, b-Fisher's, c-Mann-Whitney Wilcoxon					

Moreover, 20 patients in this study received both chemotherapy and immunotherapy, and six patients (33.33%) experienced recurrences, with no significant differences in recurrence rates or timing between neoadjuvant and adjuvant groups (p = 0.27 and p = 0.32, respectively), as shown in Table 6. 

**Table 6:** Clinical outcomes of chemotherapy cases with immunotherapy (Neoadjuvant vs Adjuvant) b-Fisher's Exact, c-Mann-Whitney Wilcoxon. IQR: Interquartile range. *Patients with missing documentation regarding that specific category are excluded from the percentage calculation for that category.

Outcome	Total (n=20)	Neoadjuvant + Immunotherapy (n=14)	Adjuvant + Immunotherapy (n=6)	p-value
Recurrence, n (%)* * * *				
Yes	6 (33.33%)	3 (23.08%)	3 (60.00%)	0.27b
No	12 (66.67%)	10 (76.92%)	2 (40.00%)	
Missing	2 *			
Recurrence pattern, n (%)* *				
Local	2 (33.33%)	0 (0.00%)	2 (66.67%)	0.40b
Regional	0 (0.00%)	0 (0.00%)	0 (0.00%)	
Distant	3 (50.00%)	2 (66.67%)	1 (33.33%)	
Multiple	1 (16.67%)	1 (33.33%)	0 (0.00%)	
N/A	14			
Time to recurrence (months)* *				
Median (IQR)	9 (5, 12)	12 (6, 33)	5 (1.5, 12)	0.32c
Range	1.5-33	6-33	1.5-12	

**Figure 1:**
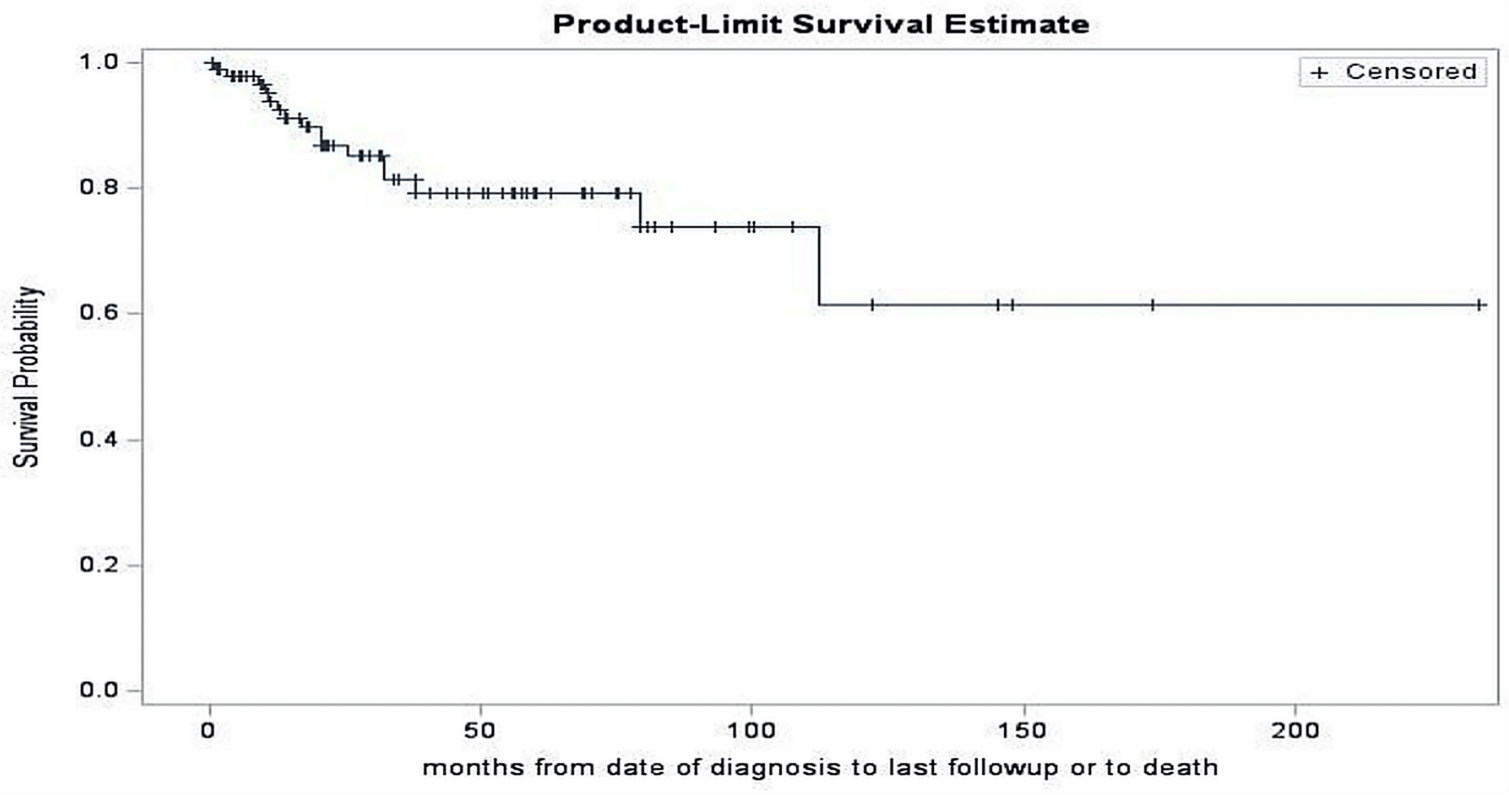
Product-limit survival estimate Overall survival rates were 93.9% at 12 months, 81.3% at 36 months, and decreased to 79.2% at 60 months. *Censoring: Patients lost to follow-up are accounted for by not reducing the survival estimate; they are "censored" in the calculation.

**Figure 2:**
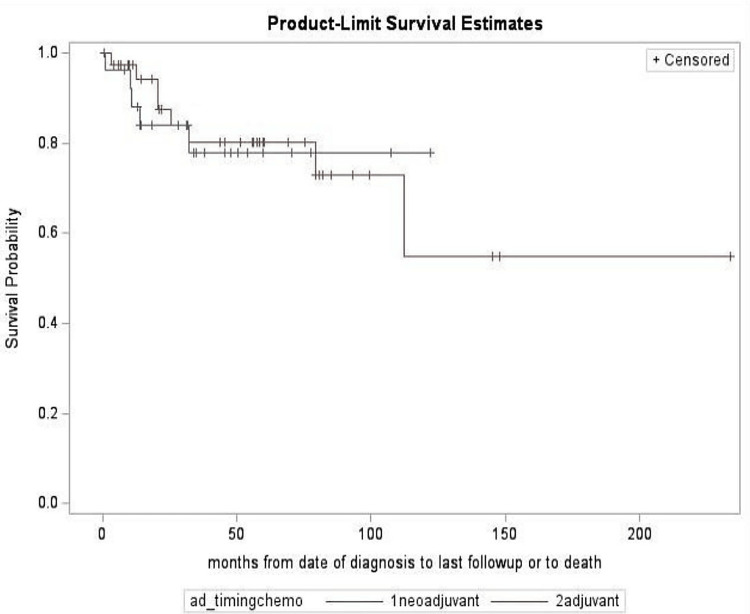
Product-limit survival estimate for chemotherapy cases The adjuvant chemotherapy group had a 60-month survival rate of 73.0% with a no estimable 60-month survival rate for neoadjuvant cases due to shorter follow-up in our study. *Censoring: Patients lost to follow-up are accounted for by not reducing the survival estimate; they are "censored" in the calculation. Blue line indicates neoadjuvant and red line indicates adjuvant

Overall survival rates were 93.9% (standard error (SE) = 2.7%) at 12 months, 81.3% (SE = 4.8%) at 36 months, and decreased to 79.2% (SE = 5.1%) at 60 months, as shown in Figure 1. There were no significant survival differences between neoadjuvant and adjuvant chemotherapy groups. The adjuvant chemotherapy group had a 60-month survival rate of 73.0% with no estimable 60-month survival rate for neoadjuvant cases. This could be attributed to the shorter follow-up in our study. The log-rank test comparing survival curves in these subgroups yielded a non-significant result (p = 0.88), as shown in Figure 2.

## Discussion

This is a single-institution retrospective review study of patients diagnosed with MtBC from 2009 to 2024. MtBC is a rare type of breast cancer that is currently not fully understood. The goal of our study was to utilize clinicopathological data to provide a comprehensive description of the characteristics of MtBC in 91 patients, specifically with an emphasis on recurrence and survival. This study supports previous literature findings that MtBC is an aggressive form of cancer. Our results showed that a majority of the tumors were predominantly high-grade (Grade 3) (n=70, 81.40%), triple-negative receptor status (ER-/PR-/HER2-), and had a high Ki-67 index. Another study evaluating the prognostic and therapeutic considerations of MtBC reported similar findings, as the majority of their patient population exhibited the TN subtype, Grade 3 tumor, and high levels of Ki-67 [10]. Utilizing these findings, there is a potential for researchers to set a standard for identifying MtBC with more ease.

For our study, the overall five-year survival rate was 79.2%. This is consistent with the previously published survival estimates for MtBC [11]. Recurrence occurred in 29 patients (36%), most of whom presented with distant metastases or multifocal disease. Based on a chart review, multiple patients had lung metastases seen on CT scans, which may indicate a possible hematogenous spread, as mentioned in previous studies [12]. We hypothesize that chest tomography will play a crucial role in the patient's one-year follow-up appointment, as the median recurrence rate occurs within the first year. The squamous cell carcinoma histologic subtype had the most frequent recurrence, while the adenosquamous subtype had no recurrence, indicating a possible subtype-specific recurrence behavior that requires further investigation.

Due to the limited knowledge and absence of treatment guidelines, patients in this study received variable chemotherapy regimens. Of the patients who received NAC therapy, seven had a complete pathological response, in which two patients received the keynote-522 clinical algorithm. This further signifies the importance of keynote-522 and supports the published case reports of complete pathological response with the keynote-522 clinical algorithm [13]. While these findings are encouraging, our study revealed no statistically significant difference in recurrence rates between the NACT and adjuvant chemotherapy groups (p=0.93), as the recurrence rates were very similar between the two groups (36.0% (n=9) vs. 37.1% (n=13), respectively). Additionally, survival curves were statistically identical (log-rank p = 0.88), suggesting a comparable outcome. The recurrence pattern and time to recurrence were also not different. These findings highlight the need to investigate NACT in a larger population, while also assessing the effectiveness of the keynote-522 algorithm. 

We attempted to identify clinicopathologic characteristics that might explain the variability in recurrence rate observed within the NACT group. However, based on the subgroup analysis, there were no significant differences in clinicopathological characteristics between NACT patients who did and did not experience recurrence. This may be attributable to the uniformly aggressive features of MtBC observed in our study population.

A group of 20 patients within our cohort received both chemotherapy and immunotherapy. This subgroup was divided into immunotherapy with NACT and immunotherapy with adjuvant chemotherapy. There were no statistically significant differences in recurrence rates, recurrence patterns, or time to recurrence between those who received immunotherapy in the neoadjuvant versus adjuvant setting. Given the limited sample size, further investigation into the role of immunotherapy is recommended to spot statistically significant differences. One case study that utilized immunotherapy in conjunction with chemotherapy for MtBC treatment reported that the dual therapy could provide long-term clinical benefits; however, future trials would still be needed to definitively confirm this [14].

Despite the uniformity of the aggressive clinicopathological characteristics of MtBC, subgroup analyses highlight the possible heterogeneity of this cancer's behaviour. The small number of patients in the subgroup might have limited the detection of clinically relevant differences. However, this study is a starting point for a database collection of MtBC cases, as it aims to prompt further investigation into the subgroup characteristics of MtBC in a larger cohort.

## Conclusions

MtBC exhibits aggressive oncologic behaviour, commonly with triple-negative receptor status, high Ki-67, and high grade. The distant metastatic pattern of recurrence was more common, indicating an aggressive nature of the disease. As a result, it is crucial to administer additional imaging, mainly to rule out lung metastases. The pathological complete response rate with NACT use was also intriguing. Further studies investigating the effects of NACT, especially keynote-522, in a larger population are necessary to create a standardized treatment plan.
